# Drug resistance and combating drug resistance in cancer

**DOI:** 10.20517/cdr.2019.10

**Published:** 2019-06-19

**Authors:** Xuan Wang, Haiyun Zhang, Xiaozhuo Chen

**Affiliations:** ^1^Department of Biological Sciences, Ohio University, Athens, OH 45701, USA.; ^2^Interdisciplinary Graduate Program in Molecular and Cellular Biology, Ohio University, Athens, OH 45701, USA.; ^3^The Edison Biotechnology Institute, Ohio University, Athens, OH 45701, USA.; ^4^Department of Chemistry and Biochemistry, Ohio University, Athens, OH 45701, USA.; ^5^Department of Biomedical Sciences, Heritage College of Osteopathic, Ohio University, Athens, OH 45701, USA.

**Keywords:** Cancer stem cells, epithelial mesenchymal transition, ATP binding cassette transporters, extracellular ATP, macropinocytosis, epigenetics, microRNA

## Abstract

Cancer is the second leading cause of death in the US. Current major treatments for cancer management include surgery, cytotoxic chemotherapy, targeted therapy, radiation therapy, endocrine therapy and immunotherapy. Despite the endeavors and achievements made in treating cancers during the past decades, resistance to classical chemotherapeutic agents and/or novel targeted drugs continues to be a major problem in cancer therapies. Drug resistance, either existing before treatment (intrinsic) or generated after therapy (acquired), is responsible for most relapses of cancer, one of the major causes of death of the disease. Heterogeneity among patients and tumors, and the versatility of cancer to circumvent therapies make drug resistance more challenging to deal with. Better understanding the mechanisms of drug resistance is required to provide guidance to future cancer treatment and achieve better outcomes. In this review, intrinsic and acquired resistance will be discussed. In addition, new discoveries in mechanisms of drug resistance will be reviewed. Particularly, we will highlight roles of ATP in drug resistance by discussing recent findings of exceptionally high levels of intratumoral extracellular ATP as well as intracellular ATP internalized from extracellular environment. The complexity of drug resistance development suggests that combinational and personalized therapies, which should take ATP into consideration, might provide better strategies and improved efficacy for fighting drug resistance in cancer.

## Introduction

Cancer is the second leading cause of death in the US^[1]^. In 2017, about 1.7 million people were diagnosed with cancer and 0.6 million people died from the disease^[2]^. Drug resistance and the resulting ineffectiveness of the drug treatment are responsible for up to 90% of the cancer related deaths^[3-7]^.

Drug resistance in cancer is a well-known phenomenon that results when cancer becomes tolerant to pharmaceutical treatment. Resistance to anticancer drugs arises from a wide variety of factors, such as genetic mutations and/or epigenetic changes, conserved but upregulated drug efflux, and various other cellular and molecular mechanisms.

Current major treatments for cancer management include surgery, cytotoxic chemotherapy, targeted therapy, radiation therapy, endocrine therapy and immunotherapy^[8-12]^. Despite the achievements made in treating cancers during the past decades, resistance to classical chemotherapeutic agents and/or novel targeted drugs continue to be a major problem in cancer therapies and responsible for most relapses, one of the major causes of death in cancer^[3-7]^. Many classical chemotherapeutic anticancer agents kill cancer cells by directly damaging their DNA, which has the problem of non-specificity and relatively high toxicity. In recent decades, more and more targeted drugs have been developed to precisely target/block changes that drive cancer growth and proliferation. Although these drugs show remarkable effects during initial treatment, a large majority of patients develop resistance as treatment proceeds. For example, 30%-55% of patients with non-small cell lung cancer (NSCLC) relapse and die from the disease afterwards^[13]^. The 50%-70% of ovarian adenocarcinomas reoccur within 1 year after surgery and associated chemotherapy^[14]^. About 20% of pediatric acute lymphoblastic leukemia patients develop recurrence^[15]^.

Better understanding of mechanisms underlying the acquisition of drug resistance is urgently needed and will facilitate developing novel therapeutic strategies and lead to better clinical outcomes. In this review, we will describe intrinsic and acquired drug resistance, present new discoveries in specific mechanisms of drug resistance and discuss new strategies for fighting against drug resistance and improving anticancer efficacy.

## Intrinsic and acquired drug resistance

Drug resistance can be categorized as intrinsic or acquired resistance based on the time when it is developed. Intrinsic resistance exists before drug treatment while the acquired resistance is induced after therapy, each occurring in about 50% of cancer patients with drug resistance^[16,17]^.

### Intrinsic resistance

Intrinsic resistance is usually defined as the innate resistance that exists before the patient is administered with (exposed to) drugs, which usually causes reduced efficacy of the drug treatment. Intrinsic resistance can be caused by: (1) pre-existing (inherent) genetic mutations in a majority of tumors that result in decreased responsiveness of cancer cells, such as triple negative breast cancer cells, to both chemo and target drugs; (2) heterogeneity of tumors in which pre-existed insensitive subpopulations, including cancer stem cells, will be selected upon drug treatment thus leading to relapse in later stages of therapeutic treatment; (3) activation of intrinsic pathways used as defense against environmental toxins (such as anticancer drugs).

Intrinsic drug resistance may exist in cancer cells prior to therapies due to the presence of genetic mutation(s) of genes involved in cancer cell growth and/or apoptosis. For example, poorer outcome of cisplatin treatment in gastric cancer patients was found to be associated with HER2 overexpression^[18]^. The high expression of *HER2* gene promoted the upregulation of transcription factor Snail, inducing a morphological change similar to epithelial-mesenchymal transition (EMT), which renders cancer cells more resistant. Furthermore, the HER2/Snail double positive patients were found to have lower survival rate than patients with only single positive or double negative genetic makeup.

It was shown that transcriptional repressors Snail and Slug not only mediate EMT, but also resistance to p53 induced apoptosis, and a self-renewal program^[19]^. The latter two activities make cancer stem cells (CSCs) more resistant to radiation and chemotherapies. It was also shown that resistant cells are more mesenchymal-like^[20,21]^. These concomitant changes link intrinsic drug resistance with EMT and CSCs.

Pre-existing resistant subpopulations in tumors may also lead to relapse after chemotherapeutic treatment. Increasing evidence shows that existence of intratumoral genetic heterogeneity in primary tumors predates clinical intervention^[22-24]^. Patients would respond to therapy initially because the majority of the tumor cells are sensitive to the drug. However, the resistant subclones would proliferate after the drug treatment and cause recurrence^[25-27]^. This intrinsic drug resistance is sometimes mistaken for acquired resistance since the tumor would shrink first upon treatment and the resistance seems to be acquired due to therapy. CSCs are a subpopulation in tumors with the capacity of self-renewal and differentiation, participating in tumor initiation and progression^[28]^. They have been reported to be involved in resistance to chemotherapeutic drugs in multiple cancer types, including leukemia^[29]^, glioblastoma^[30]^ and pancreatic cancer^[31]^. Combined therapy targeting both CSCs and most tumor cells might be required to reduce drug resistance.

Therapeutic effects of a drug can be reduced by activation of intrinsic pathways that are used as a defense against environmental toxins, including anticancer drugs. Examples of these protective mechanisms include ATP binding cassette (ABC) transporter mediated drug efflux^[32]^ and glutathione (GSH)/glutathione S-transferase system^[33]^, working to reduce cellular drug accumulation or detoxify drug treated cancer cells respectively.

### Acquired resistance

Acquired resistance can be identified by gradual reduction of anticancer efficacy of a drug after the drug treatment. Acquired resistance can be a result of: (1) activation of second proto-oncogene that becomes the newly emerged driver gene; (2) mutations or altered expression levels of the drug targets; (3) changes in tumor microenvironment (TME) after treatment.

Shrunk tumors can acquire resistance and regrow due to new mutations. In one study, genomic profiles before and after relapse of eight acute myeloid leukemia patients were analyzed using whole-genome sequencing^[34]^. The comparison of mutations between primary and relapse tumors revealed novel gene mutations. In addition, the results showed increased transversion mutations in relapsed tumors, suggesting that cytotoxic chemotherapeutic drugs caused DNA damage in cancer cells and might increase probability of the emergence of new mutations.

Cancer cells can acquire resistance to targeted drugs when the genes encoding target proteins develop new mutations or change expression levels. One example of secondary mutations within the target kinase is the threonine 315 to isoleucine (T315I) mutation in the BCR-ABL kinase domain. Tyrosine kinase inhibitor (TKI) imatinib targeting BCR-ABL is commonly used in chronic myelogenous leukemia treatment, but approximately 20%~30% of patients will experience resistance or relapse after treatment^[35]^. One mechanism of the resistance is due to a point mutation of T315I of the fusion tyrosine kinase protein^[35-37]^. The change of threonine 315 to isoleucine results in a loss of a hydrogen bond, which is necessary for the binding of imatinib to the ATP-binding site of BCR-ABL, leading to significantly reduced efficacy of the drug.

Drug resistance can also be acquired due to the dynamic changes of TME in the course of treatment. During malignancy progression and development of resistance, cross-talk exists between tumor cells and their microenvironment. Exosomes released by cancer and stromal cells are involved in the crosstalk. Researchers found that exosomes, which are released by cancer cells and carry certain miRNAs were used by cancer cells and tumor-associated macrophages (TAMs) in the TME to communicate with each other^[38]^. In cisplatin treated neuroblastoma (NBL) tumor, NBL cells secret exosomic miR-21 to induce TAMs to produce exosomic miR-155 to in turn silence the *TERF1* gene in NBL cells. TERF1 protein is an inhibitor of telomerase, and decreased expression of TERF1 would result in increased telomerase activity and resistance to chemotherapy. Therefore, the exchange of exosomic miRNAs between tumor cells and stromal cells in TME can promote drug resistance.

Mechanisms of intrinsic and acquired resistance described above can co-exist during tumor progression and treatment. Mechanisms of acquired drug resistance can be totally different from the pre-existing intrinsic drug resistance. Alternatively, it can be a selective expansion of the intrinsic drug resistance.

The degree of intrinsic drug resistance pre-determines the sensitivity of given cancer cells to a specific drug. Genomic and other biochemical analyses should be performed before the drug treatment plan is designed to avoid potential pre-existing drug resistance. After acquired drug resistance is developed, therapeutic schemes need to be adjusted accordingly.

One of the objectives of drug treatment should be to effectively slow down or stop tumor growth without inducing acquired, or at least uncontrollable acquired drug resistance. The best drug treatment plan should take prevention or delay of acquired drug resistance into consideration.

## Mechanisms of drug resistance

Although it is scientifically important to distinguish intrinsic and acquired resistances, the specific mechanisms of resistance are more clinically significant.

### Increased efflux of drugs

Elevated efflux of anticancer agents, which leads to decreased intracellular drug accumulation, has been considered to be the major reason for chemotherapy resistance^[7,39,40]^. The resistance caused by the abnormally high rates of drug efflux could be either intrinsic or acquired, depending on if it exists prior to or develops after drug administration.

Transmembrane transporters responsible for the drug efflux are primarily from the ABC transporter superfamily. The human genome contains 48 *ABC* genes and they are classified into seven subfamilies (ABCA-ABCG)^[41,42]^. Among them, ABCB1, ABCC1 and ABCG2 are highly involved in the acquisition of multidrug resistance (MDR) to cancer chemotherapeutics.

ABCB1 (MDR1 or P-gp) is one of the most well-characterized ABC transporters. It is composed of two transmembrane domains that form a passage for substrates and two nucleotide-binding domains that bind and hydrolyze ATP. The binding and the subsequent hydrolysis of ATP is coupled with conformational changes in the transporter, leading to the pumping out of the transport substrates^[43]^. ABCB1 has multiple drug binding sites that can bind and pump a wide variety of substrates from the cell, such as etoposide, doxorubicin, paclitaxel and vinblastine^[44-48]^. High expression level of ABCB1 has been observed before chemotherapy in many different tumor types, including kidney, lung, liver, colon and rectum^[49]^. In contrast, initially low expression and then dramatic increased expression of ABCB1 post chemotherapy were observed in many hematological malignancies, such as AML and ALL^[50-52]^.

ABCC1, or multidrug resistance-associated protein 1 (MRP1), similar to ABCB1, is also responsible for pumping out a wide variety of anticancer agents, such as vinca alkaloids, anthracyclines, epipodophyllotoxins, camptothecins, and methotrexate^[53]^. While ABCB1 transports amphipathic and lipid-soluble compounds, ABCC1 pumps organic anionic substrates such as compounds conjugated to glutathione, glucuronide, or sulfate^[54-56]^. Overexpression of ABCC1 has been shown to be associated with resistance in many cancer types including lung, breast and prostate cancers^[53,57,58]^.

ABCG2, or breast cancer resistance protein, is the major drug efflux transporter in breast cancer associated resistance, as indicated by the name. ABCG2 is considered a marker of CSCs in some cancers, and responsible for the side-population effect. It can transport both positively- or negatively-charged drugs, ranging from chemotherapeutic drugs (mitoxantrone, bisantrene, epipodophyllotoxin, camptothecins, flavopiridol and anthracyclines) to several TKIs (imatinib and gefitinib)^[48,59,60]^. Besides breast cancer, ABCG2 overexpression was also found in many other cancer types including lung cancer and leukemia^[60,61]^.

Other ABC transporters have also been studied for their substrates and functions in tumor resistance to anticancer drugs, providing additional explanations on the mechanisms of drug resistance^[62]^. For example, ABCC2 and ABCC3 can transport many chemotherapeutic drugs, including cisplatin, doxorubicin, and etoposide, and their overexpression results in multidrug resistance^[62-65]^. Mutations and overexpression of ABC transporters directly influence tumor sensitivity and drugs’ anticancer efficacy. An accurate and complete expression profile of ABC transporters in tumors is important for proper drug selection and better treatment outcomes.

### Alteration of drug target

Compared to traditional chemotherapies which kill cancer cells by disrupting rapid cell proliferation and may affect normal dividing cells, targeted therapies can block the growth of cancer cells by inhibiting the activity of specific target proteins involved in tumor development, thus being more selective and effective to cancer cells and less harmful to normal cells. However, targeted therapy may also develop the problem of resistance, resulting from alteration of drug targets. The alteration of drug targets may be either a secondary mutation in the target protein or changes in expression levels due to epigenetic alterations.

For example, TKIs of the epidermal growth factor receptor (EGFR), such as erlotinib and gefitinib targeting NSCLC, have been reported to show high response rate at initial treatment^[66,67]^. However, almost 50% of the responsive patients would develop a T790M mutation on EGFR within one year, resulting in resistance to the first and second generations of TKIs^[68-70]^. The mutation from threonine to methionine led to a configuration change in EGFR and consequently enhanced ATP binding affinity and impaired binding of gefitinib/erlotinib for the kinase^[70,71]^. To overcome the resistance caused by T790M, third generation of TKIs, like osimertinib and rociletinib, has been developed and reported to show clinical efficacy with patients harboring T790M mutation^[72,73]^. However, resistance to third-generation inhibitors develops not long after their use, raising the need of developing fourth generation TKIs. One reported major mechanism of the new resistance is due to a mutation in EGFR known as C797S^[74]^. The loss of the cysteine residue, which is important for TKIs to target the ATP site, impairs the binding of the third generation TKIs to EGFR. Therefore, EAI045, a fourth generation TKI targeting both T790M and C797S, has been designed to bind an allosteric site located on EGFR, attempting to circumvent the mechanism patterns of resistance to the early generations of TKIs which all bind to the ATP sites^[75,76]^. The battle between generation of new genetic mutations and generation of new TKIs that restore drug sensitivity may become a new trend in the everlasting war against drug resistance.

The development and use of estrogen receptor inhibitors in breast cancer treatment provide another example for the resistance induced by alteration of the drug target. Tamoxifen (TAM) is commonly used for patients with ER-positive breast cancer, relying on its ability to compete with estrogen for the ligand binding site of ER. However, extended exposure of TAM often leads to drug resistance. Mechanisms of resistance vary in different cases, and mutations in the ER gene and decrease in ER expression level are among them^[77,78]^. Given the problems with TAM and the quest for alternative drugs, aromatase inhibitors (AIs) were developed, working by interfering with the last step of estrogen synthesis. Third-generation AIs are now being used as first-line therapy in postmenopausal women with hormone receptor positive breast cancer^[79]^.

### Enhanced DNA damage repair

Many chemotherapy drugs, like cisplatin and 5-fluorouracil (5-FU), kill cancer cells by inducing DNA damage. The DNA damage response (DDR) of affected cells to the anti-cancer drugs may result in reduced efficacy of the drugs by DNA lesion repairs, leading to drug resistance^[80]^. For example, genes involved in DNA repair, like FEN1, FANCG, RAD23B, were found to be upregulated in 5-FU resistant human colon cancer cell lines^[81,82]^. 5-FU treatment induced upregulation of p53-target genes on DNA damage response and repair. Success to repair the damages led to reduced cell cycle arrest and apoptosis in the resistant cell lines compared to parental cell lines^[82]^.

Although deregulation of DDR may remit the resistance induced by DNA repair, it may also increase the risk of developing new mutations due to genomic instability, the accumulation of which may initiate a new round of carcinogenesis. Therefore, DNA damage response is a complex mechanism in cancer treatment and recurrence, and it requires thorough consideration when used as an anticancer therapeutic target.

### Senescence escape

Cellular senescence refers to irreversible arrest of cell proliferation, largely leading to activation of tumor suppressive pathways mediated by p53 and/or p16^INK4a[83]^. Cellular senescence can be triggered by endogenous and exogenous stimuli, among which the three major stimuli include excessive mitogenic signaling produced by activated oncogenes, telomere shortening^[84]^, and non-telomeric DNA damage caused by chemotherapeutic drugs. For example, Doxorubicin and Cisplatin used in chemotherapy by inducing cell death can also initiate senescence^[85,86]^.

Escape from therapy-induced senescence (TIS) has been recognized as a mechanism for drug resistance and tumor recurrence/progression^[87]^. Cancer cells with TIS can gain stem-cell properties, which accounts for the escape from senescence and cancer relapse^[88,89]^.

### Epigenetic alterations

An emerging mechanism contributing to drug resistance is epigenetic alterations. Increasing evidence brought people’s attention to epigenetic modifications that also participate in the development of other mechanisms of resistance, including increased drug efflux, enhanced DNA repair, and impaired apoptosis.

Epigenetic modifications include DNA methylation, histone modification, chromatin remodeling, and non-coding RNA related alterations. For example, demethylation of DNA at the promoter region of an oncogene would upregulate the expression of the gene, resulting in drug resistance. A recent study demonstrated that a G-actin monomer binding protein thymosin β4 (Tβ4) was enriched through demethylation of DNA and active modification of histone H3 at the promoter region in a resistant hepatocellular carcinoma (HCC) cell line^[95]^. Overexpression of Tβ4 led to the acquisition of stem cell-like capacity in the HCC cell line and induced resistance to VEGFR inhibitor sorafenib *in vivo*^[95]^.

Besides chromosomal modification, non-coding RNAs, including microRNAs (miRNAs) and long non-coding RNAs (lncRNAs), also play an important role in drug resistance^[96,97]^. MiRNAs contain about 21-25 nucleotides (nt) while lncRNAs can range from 200 to more than 10,000 nt in length. MiRNAs are known to be important regulators of post-transcriptional gene expression by binding to their complementary mRNAs and mediating mRNA degradation and repression of protein synthesis. LncRNAs participate in gene expression regulation in different ways such as blocking the binding of transcription activators to key DNA sequences in genes or recruiting chromatin remodeling proteins. Both miRNA and lncRNA regulate expression of proteins related to cancer drug resistance. For example, lncRNA urothelial cancer-associated 1 (UCA1) was shown to be upregulated in cisplatin-resistant bladder cancer cells compared to sensitive cells^[98]^. Upregulation of UCA1 expression resulted in significantly increased mRNA and protein levels of wingless-type MMTV integration site family member 6 (Wnt6), promoting Wnt signaling and cell survival^[98]^.

### Tumor heterogeneity

Four levels of heterogeneity are present in tumors: genetic heterogeneity, cell type heterogeneity (cancer cells, stromal cells, immune cells, *etc*.), metabolic heterogeneity in oxygen/nutrient distribution, and temporal heterogeneity in dynamic tumor progression^[99]^. Tumor heterogeneity increases the complexity and difficulty of cancer treatment, making it almost impossible to kill all cancer cells using one single therapeutic. This problem led to the development of combinational therapies used in many cancer treatments such as FEC: 5-FU, epirubicin, cyclophosphamide for breast cancer. In this section, genetic heterogeneity will be the focus of discussion.

Ample evidence has shown that subpopulations of cancer cells with various genetic makeups co-exist in primary tumors like ovarian cancer^[100]^, renal cell carcinoma^[101]^, breast cancer^[102]^, and chronic lymphocytic leukemia^[103]^. These clonal variants have different sensitivity to chemo or targeted drugs, so that initial treatment can kill only a portion of the tumor and those less sensitive cancer cells would survive. Once the resistant clones proliferate and grow, the tumor would come back with different cell composition portions that are insensitive to the initial chemotherapy. This genomic heterogeneity of the subpopulations evolves under drug treatment in a Darwinian selection manner, supported by evidence that subclonal compositions differ significantly at different stages of treatment^[23,24,101,104]^. Also, in heterogeneous populations of tumor cells, drug-resistant tumor cells can transfer microRNAs by exosome to drug-sensitive tumor cells and induce resistance to the latter^[105]^.

The contribution of tumor heterogeneity to drug resistance is supported by studies reporting the loss of drug sensitivity to targeted drugs. The high specificity of targeted therapies, which is their advantage in increasing efficacy and reducing side effects, may become a limitation when dealing with tumor heterogeneity. Therefore, combined/cocktail therapies with more than one drug are required to overcome or delay the relapse of tumor.

Furthermore, heterogeneity among patients would result in differences in the patients’ response to the same treatment, urgently calling for the development of individualized therapies.

### TME

Tumors are not bags of homogeneous cancer cells, but contain various types of cells and extracellular matrix (ECM) that work together to contribute to all aspects of the hallmarks of cancer^[106,107]^. Microenvironment of solid tumors includes ECM, immune and inflammatory cells, blood vessels, fibroblasts, and various nutrients and signaling molecules. They work in a coordinated manner to play vital roles in tumor growth and survival.

TME may contribute to intrinsic resistance to anticancer therapies. One of the TME factors is pH. In normal tissues and cells, extracellular pH is usually slightly higher than intracellular pH (pHe7.3-7.5 *vs.* pHi6.8-7.2)^[108]^. In contrast, cancer cells develop a so-called “reversed pH gradient”, with increased intracellular pH and decreased extracellular pH, through the proton pumping of proton transporters and the modulation of pH sensors^[109,110]^. The acidic (pH6.5-7.1) extracellular environment of cancer cells has been reported to be a contributor to the resistance to chemotherapeutics^[111]^. The reversed pH gradient impairs the distribution of weak base anticancer drugs, which is described as “ion trapping”, and enables cancer cells to evade apoptosis^[112,113]^. As an emerging hallmark of solid tumors, the low extracellular pH would be a potential target for cancer therapy. Therapeutic approaches attempting to reduce microenvironment acidity, such as proton pump inhibitors (PPIs), have been developed and shown good efficacy in shrinking tumor and sensitizing cancer cells to chemotherapy drugs. Lansoprazole, an example of PPI, has been reported to demonstrate synergistic effects when used in combination with paclitaxel in melanoma cells both *in vitro* and *in vivo*^[114]^.

Changes in the composition of TME after treatment also contribute to the adaptation of cancer cells to chemo or targeted therapeutics, thereby reducing drug efficacy and inducing resistance. For example, TAMs play a role in the acquisition of resistance in response to anticancer therapies in glioblastoma multiforme (GBM), a severe type of brain tumor^[115]^. Macrophages secrete high levels of colony stimulating factor-1 (CSF-1) in GBM tumors, supporting cancer cell proliferation and survival^[115,116]^. Therefore, CSF-1 receptor (CSF-1R) has been targeted by small molecule inhibitors or antibodies in cancer treatment with promising *in vivo* effects^[117-119]^. However, more than 50% GBM patients suffer from recurrence, which is derived from elevated secretion of insulin-like growth factor-1 (IGF-1) from TAMs and IGF-1 induced elevation of phosphatidylinositol 3-kinase (PI3K) pathway signaling in GBM tumor cells^[115]^. Combining inhibition of CSF-1R with inhibition of IGF-1 receptor or PI3K signaling has been reported to extend overall survival in mouse models^[115]^. Thereby, combined therapies simultaneously targeting cancer cells and TME may produce much improved anticancer efficacy by reducing drug resistance.

Besides TME heterogeneity per se being one aspect of tumor heterogeneity, it also contributes to the enrichment of genetic heterogeneity. For example, due to variation and dynamic nature of vasculature inside tumors, fluctuating hypoxia is one characteristic of TME^[120]^. The frequent cycles of hypoxia and reoxygenation produce oxidative stress that could induce DNA damages in tumor cells, thus contributing to genetic instability that leads to accumulation of additional mutations and emergence of genetically divergent clonal subpopulations^[121]^. Moreover, as mentioned previously, cells in TME, like TAMs, interfere with the expression profiles of cancer cells by releasing miRNA-containing exosomes^[38]^, thus contributing to tumor heterogeneity.

Therefore, TME plays very significant role in tumor progression and therapeutic resistance. Better understanding and targeting/manipulating TME and its interaction with tumor cells could substantially enhance therapy response and achieve better clinical outcomes.

### EMT

EMT is a process during which epithelial cells lose their attachment to each other and gain the characteristics of mesenchymal stem cells. EMT has been known to be essential for the initiation of metastasis in tumors of epithelial origin, but it is not so clear about its role in other tumors such as sarcomas. Increasing evidence shows that EMT plays a critical role in chemotherapy resistance. Fischer *et al*.^[122]^ reported that EMT promotes resistance to apoptosis induction activated by drug cyclophosphamide in an EMT lineage-tracing mouse system. However, the mechanisms of EMT-induced drug resistance are not fully understood although most recent studies suggest that EMT and CSC share some similarities and their involvements in drug resistance represent different manifestations of the same phenotype. One possible mechanism is that EMT cells share many similarities in signaling pathways with cancer stem cells (CSCs), such as Wnt, Notch and Hedgehog pathways^[123]^. Therefore, EMT enables tumor cells to gain resistance to anticancer drugs and evade drug induced cell death. For example, TGF-β is a well-studied key cytokine in EMT, the signaling pathways of which are correlated with gain of drug resistance^[124,125]^. Inhibition of TGF-β can reverse the process of EMT and remarkably increased the sensitivity of cancer cells to chemotherapies^[126,127]^. Wnt and Hedgehog pathways are also reported to be related with drug resistance^[128,129]^.

Furthermore, accumulating evidence shows that the CSCs rely on the EMT program as a critical regulator when mediating drug resistance. The CSC state of carcinoma cells requires epigenetic changes resulted from activation of EMT. Understanding the mechanistic linkage between EMT, CSC and drug resistance would significantly contribute to anticancer therapeutics^[130]^.

EMT inducing transcriptional factors (EMT-TFs) also play roles in promoting drug resistance. Overexpression of EMT-TFs like Twist, Snail, Slug, ZEB and FOXC2 are known to induce drug resistance^[131-135]^. One recent study reported that suppressing EMT by knocking out EMT transcription factors Twist1 or Snail1 enhanced sensitivity to gemcitabine and increased survival rate in pancreatic ductal adenocarcinoma-bearing mice treated with the drug^[136]^. Some of these EMT-TFs promote resistance by enhancing drug efflux by ABC transporters. Promoters of genes coding for ABC transporters were found to have EMT-TF binding sites^[137]^. Overexpression of Twist, ZEB1/2, Slug, and Snail enhances expression and activity of ABCB1, thus inducing drug resistance^[138-140]^. ABCG2, another ABC transporter closely linked to MDR, is known to be regulated by Snail, MSX2, SOX2 and ZEB1^[141-144]^. Other ABC transporters involved in MDR, such as ABCC1, ABCC2, ABCC4, and ABCC5, also have been shown under the regulation of EMT-TFs^[145-147]^. For example, overexpression of ABCC5 is correlated with FOXM1 in paclitaxel-resistant nasopharyngeal carcinoma cells. Depletion of either FOXM1 or ABCC5 decreases drug efflux and increases cell death induced by paclitaxel^[148]^. Knockdown of these EMT-TFs sensitize cancer cells to chemotherapeutic drugs by suppressing ABC transporters^[142,143,148]^. Targeting these EMT-TFs may inhibit metastasis and drug resistance at the same time.

Besides EMT-TFs, miRNAs are believed to be essential molecules that link EMT and ABC transporters^[149]^. MiRNAs are small endogenous RNA of 20-24 nucleotides, which have a complex network and can regulate expression of genes that are associated with EMT and are of ABC family^[149]^. Haenisch *et al*.^[150]^ have summarized miRNA-mediated regulations of ABC transporters. MiRNA network regulates ABC transporters at different levels, with the majority of miRNAs acting at post-transcriptional levels via binding sites at the three prime untranslated region (3’-UTR) and some miRNAs acting at transcriptional levels by binding to the gene promoter region^[151]^. miRNAs can not only regulate expression of ABC transporters but also regulate EMT markers. For example, miR-200c is reported to both down-regulate multiple ABC transporters including ABCB1 and ABCG2^[152,153]^, and negatively regulate EMT by directly targeting 3’-UTR regions of ZEB1 and ZEB2, and retain the epithelial phenotype^[154]^. Some miRNAs, such as miR-200c and miR-145, inhibit ABC transporters and suppress EMT^[155]^; other miRNAs like miR-27a positively regulate ABC transporters and promote EMT^[156,157]^.

MiRNAs also regulate drug resistance by other mechanisms including regulating apoptosis and autophagy, controlling anti-cancer drug metabolism, modulating drug targets and DNA repair, and regulating GSH biosynthesis, which has been summarized by An *et al*.^[158]^. One recent study revealed that over expression of miR-134, miR-487b, and miR-655 promotes TGF-β induced EMT and drug resistance to gefitinib in NSCLC^[159]^. This miRNA cluster induces resistance to EGFR-TKI by directly inhibiting MAGI2, leading to reduced PTEN activity. Reduction of PTEN and upregulation of PI3K-Akt-pathway is associated with acquired EGFR-TKI resistance^[160]^.

**Figure 1:**
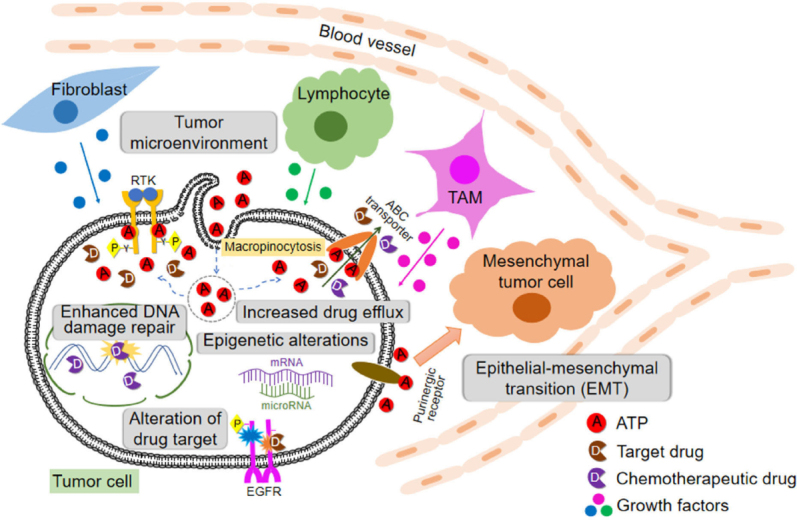
Cells, protein factors, and mechanisms involved in drug resistance in cancer described in this review including extracellular ATP-induced resistance. ABC: ATP binding cassette; RTK: receptor tyrosine kinases; EGFR: epidermal growth factor receptor; TAM: tumor-associated macrophage

All these discussed drug resistance mechanisms are schematically shown in Figure 1.

Drug resistance can be intrinsic or extrinsic in nature. Drug resistance is induced at levels of DNA (enhanced DNA damage repair and epigenetics alternations), RNA (microRNA), and proteins [concentration and activity changes of receptor tyrosine kinases (RTK in general and EGFR in specific, and also ABC transporters)]. In addition, different stromal cells and stressed/lysed cancer cells in a tumor release ATP into intratumoral space, creating a high ATP concentration TME. Intratumoral extracellular ATP (eATP) works as a messenger outside of cancer cells through purinergic signaling cascade to induce EMT, which contributes to drug resistance. eATP is also internalized by cancer cells via macropinocytosis, leading to greatly elevated intracellular ATP (iATP) levels. The higher iATP levels in turn enhance the efflux activity of ABC transporters for pumping anticancer drugs out of cancer cells, increase competition between iATP with ATP analog anticancer drugs at the intracellular ATP binding domain of RTKs located on cancer cell plasma membrane and increase RTK phosphorylation. All these mechanisms work together dependent or independent of ATP to augment drug resistance by reducing intracellular drug concentration, increasing cell survival signaling and inducing EMT (cancer stem cell-like features). More studies are needed for the final validation of ATP-mediated mechanisms of drug resistance.

## ATP and ATP-mediated drug resistance

### Intracellular ATP promotes drug resistance

ATP, either intracellular or extracellular, plays significant roles in cancer cell growth, survival and resistance. It is known that the intracellular ATP level in cancer cells is higher than that in normal tissues of the same origin, likely due to the upregulated glucose transport and aerobic glycolysis in cancer cells, a process also called the Warburg effect^[161-167]^. Furthermore, even higher intracellular ATP levels were found in acquired resistant cancer cells lines compared to their parental cell lines^[168,169]^. In one study using colon cancer cells lines, intracellular ATP levels were found to have a two-fold increase in the chemo-resistant cell lines compared to those in their drug-sensitive parental cell lines^[168]^. The authors demonstrated that ATP levels played a pivotal role in multiple drug resistance by showing that artificial delivery of ATP into the drug-sensitive cells resulted in drug resistance while depleting intracellular ATP with a glycolysis inhibitor sensitized resistant cancer cells. A study from another group also showed that intracellular ATP contributed to drug resistance to cisplatin in ovarian adenocarcinoma cells^[169]^. In contrast to the more glycolysis-related ATP elevation in the previous study, the authors showed that the resistant cell line had an increased intracellular ATP level as a consequence of enhanced mitochondrial ATP synthesis that was induced during the development of resistance. They suggested that the resistant cell line may have increased their energy storage for the purpose of protecting the cells from xenobiotics and environmental stress. These studies showed the metabolic- (energetic-) role of intracellular ATP in acquired resistance to chemotherapeutic drugs. Elevated intracellular ATP levels is likely to be a necessary condition for cancer cells particularly resistant cancer cells.

For all the reasons mentioned above, inhibitors targeting glycolysis related proteins (GAPDH and LDH) like 3-bromopyruvate, oxamate and FX11 that significantly deplete intracellular ATP levels might sensitize cancer cells to anticancer drugs^[170-174]^.

### Extracellular ATP induces drug resistance

eATP levels of various cancer types have been reported to be 10^3^ to 10^4^ times higher than those in their corresponding normal tissues^[175-178]^. The functions of this high eATP have been under investigation. In our recent study, eight anticancer drugs, including both targeted and chemotherapeutic drugs, were tested in five cancer cell lines of five different organ origins, and extracellular ATP was found to promote intracellular ATP increase and cancer cell survival in most cases^[179]^. When studying ATP promoted drug resistance to sunitinib in NSCLC A549 cells, we found that extracellular ATP can be internalized by cancer cells through macropinocytosis and other endocytic mechanisms, resulting in substantially elevated intracellular ATP levels from 150 to 200% of the original intracellular ATP concentrations^[161,179,180]^. Thus, macropinocytosis, and other endocytosis-mediated extracellular ATP internalization, and the resulting intracellular ATP level elevation is responsible, at least in part, for the observed drug resistance.

One of the possible drug resistance mechanisms induced by the internalization of extracellular ATP is that more abundant intracellular ATP molecules compete with tyrosine kinase inhibitors which are ATP competitors, for the ATP binding site located on RTKs, leading to increased phosphorylation and activation of downstream signaling pathways. Meanwhile, the internalized ATP molecules also enhance efflux of TKIs and chemo drugs by ABC transporters, resulting in decreased drug accumulation and increased cell survival. The reduced intracellular drug concentrations and elevated intracellular ATP levels further enhance the ATP binding and reduce TKI binding on RTKs, leading to even more RTK-mediated signaling and drug resistance. These were all observed in the study^[179]^, which suggested the dual roles of ATP as both an energy molecule facilitating drug efflux and signal-transduction (phosphorylation) molecule activating cell survival signaling pathways.

In the five cell lines studied, increased intracellular ATP was found to correlate with drug resistance status when the ABC transporters expressed by the cell line matched those required for the efflux of a given drug^[179]^. Extracellular ATP was also shown to alter expression levels of ABC transporters, indicating the profound effects of ATP on modulating ABC transporter activities at both transporting activity rate and transporter expression levels to potentially efflux anticancer drugs and enhance drug resistance^[179]^. Prescreening of tumors based on their ability of ATP internalization (macropinocytosis) and expression of specific set of ABC transporters will provide valuable information for the selection of proper anticancer drugs and prediction of patient’s response to therapeutics, therefore reducing drug resistance and enhancing the efficacy of drug treatment. Our lab’s findings in extracellular ATP’s roles in cancer cell growth, survival and drug resistance were reviewed in a 2018 Nature Reviews Cancer paper^[181]^.

The drug resistance observed here is intrinsic in nature and could last during later treatments. Reducing intratumoral extracellular ATP concentration and/or blocking ATP internalization may increase drug efficacy at both initial and later stages of cancer treatment.

ATP might also contribute to cancer drug resistance from outside of the cell through purinergic receptor signaling. Purinergic signaling has been known to promote cell growth and proliferation^[182,183]^. Purinergic receptors such as members of the P2X and P2Y families were reported to be involved in cancer drug resistance^[184-186]^. In one recent study, researchers demonstrated that ATP promoted resistance to chemotherapeutic drugs in colorectal cancer cells through P2Y-mediated upregulation of MRP2 and concurrent drug pumping^[187]^. Another study showed that P2X7 receptor, when activated by ATP, exhibited anti-apoptotic activity in methoxyestradiol-treated melanoma cells^[185]^. Extracellular ATP has also been found to upregulate expression of glucose transporter 1, possibly via the P2X7 induced PI3K-AKT pathway and hypoxia-inducible factor 1α-dependent signaling^[181]^. These changes are also likely to increase cancer cell survival and drug resistance. Therefore, reducing the extracellular ATP concentration could increase drug efficacy by reducing specific purinergic receptor signaling in addition to reducing ATP internalization. An ATPase, apyrase, was shown to reduce the growth of glioblastoma when injected in a rat glioma model^[187]^, suggesting the feasibility of this strategy.

Extracellular ATP also plays significant role in immunoregulation in TME and potentially affects therapeutic results. ATP can be secreted into extracellular environment by cancer cells undergoing autophagy or apoptosis induced by chemotherapy or radiotherapy, generating a chemotactic gradient around dying cells and recruiting myeloid cells through purinergic signaling^[177,188-191]^. Defects in the molecular machinery for autophagy in cancer cells or purinergic receptors in immune cells lead to poor response to stimuli that would cause immunogenically induced cell death^[189,192]^. Furthermore, immune cells in TME can release and use extracellular ATP, potentially creating an ATP-rich and tumor-friendly environment. For example, lymphocytes are known to release large amounts of ATP as a signaling molecule into the extracellular space when stimulated, serving as a messenger in cellular interactions of T lymphocytes^[193]^. Stimulated monocytes can release ATP as an autocrine signal molecule^[194]^. ATP released from these immune cells as well as dying or stressed cells can be sequentially converted to AMP and adenosine by cell surface enzymes CD39 (ecto-nucleoside triphosphate diphosphohydrolase 1, E-NTPDase1) and CD73 (ecto-5’-nucleotidase, Ecto5’NTase)^[195,196]^. Adenosine mediated signaling would lead to the establishment of an immunosuppressive environment^[197,198]^. Combined administration of an inhibitor of extracellular ATPase and a synthetic TLR4 ligand was reported to restore the infiltration of necroptosis-deficient tumors by APCs and CD8^+^ T cells, and re-establish normal sensitivity to mitoxantrone-based chemotherapy^[199]^. These findings suggest that extracellular ATP is essential to anti-tumor immune response while its hydrolysis products contribute to immunosuppression. Therefore, the anti-resistance strategy through ATP degradation by ATPase is perhaps not appropriate to use together with therapies designed to boost immune system. However, this may be an extracellular ATP concentration-dependent phenomenon. There will be a concentration window for extracellular ATP where extracellular ATP concentrations are sufficiently low not to generate significant drug resistance but sufficiently high to maintain anti-tumor immune responses. Significantly more studies need to be done before we fully understand the different functions and different concentrations of extracellular ATP on cancer and immune cells in tumors.

## Conclusion: strategies for fighting against drug resistance

Due to the high heterogeneity among tumors growing in patients and high complexity of evolution of tumor progression, identifying the best strategy to overcome drug resistance will be very challenging. On the other hand, with the development of high throughput cancer genomics, cancer proteomics, and cancer metabolomics analyses, it is now possible to identify driver genes and major components that contribute the drug resistance the most at any specific stage of tumorigenesis in a patient. Due to the nature of individual differences in mutations in multiple and different cancer-causing genes, combinational and personalized therapies are required. Combinational therapies are strongly preferred since tumors are almost always multi-clonal and genetically heterogeneous. Therapeutic strategies using single drugs are most likely to lead to eventual treatment failure due to drug resistance as the treatment kills sensitive cancer cells but leaves resistant cancer cells to survive and proliferate. In comparison, combinational therapy using two or more drugs is likely to target multiple driver genes simultaneously, not only inhibiting more clones in a tumor but also making new cancer mutations resistant to multi-drug treatment much more difficult to be selected and grow up.

Current strategies to deal with drug resistance depend on continuous monitoring of patients and treatment with a cocktail of chemotherapeutic/target drugs, each targeting one or more proteins encoded by driver genes responsible for drug resistance pathways operating in cancer patients. Like recent trends of successful target drug therapy, simultaneous multi-targeting will be more effective in combating drug resistance, thereby enhancing anticancer efficacy of therapies and prolong patients’ lives. However, the outcomes of therapies inevitably depend on the composition/unique resistance profile of tumors and the toxicity tolerance of patients, so that the therapeutic results are hard to predict. Since tumor cells could always develop alternative mechanisms to circumvent current therapy, fighting against drug resistance seems to be an endless game.

Conventionally, cancers were treated with chemo or targeted drugs at the highest dosage that the patients can tolerate. In recent years, it was realized that such a treatment strategy may lead to drug resistance more rapidly, since such treatment puts constant pressure on tumors to select those cancer cells that are strongly resistant to the drugs. New treatment strategies of “on and off” or “high dose followed by low dose” resulted in longer survival and delayed drug resistance, because this intermittent or adaptive dosing may interrupt the growth of drug-dependent resistant cells and allow the competition of sensitive and resistant cells^[200]^. In one study, researchers found that melanoma cells that acquired resistance to combined BRAF- and MEK- targeted therapy displayed robust drug addiction and were exquisitely sensitive to acute drug withdrawal^[201]^. This drug sensitivity is also detected in other cancer types such as lymphoma cells treated with an ALK kinase inhibitor that became dependent on it, suggesting that intermittent dosing may prolong control of ALK+ tumors^[202]^. These findings encouraged testing of this pulsatile dosing regimen in clinical trials (ClinicalTrials.gov Identifiers: NCT02196181).

However, these new treatment strategies produced inconsistent results in that improved survival was achieved in some cases but no change or even worse outcomes occurred in others. These indicate that the new strategies only work in some specific cases but cannot be used as a general strategy. We still need to know more about drug-cancer interactions at a personalized level before we can develop treatment plans that fit individual needs.

One possible strategy of circumventing resistance is to block energy supply of tumor cells. Tumor might bypass any mechanism, but could not evade the need for energy to support their growth, proliferation and other activities such as drug resistance and cell migration. Combinational therapy with an addition of blockage of energy may increase efficacy of therapeutic reagents. Although normal cells are more versatile and flexible in using various energy supply molecules, cancer cells appear to be more rigid in using energy sources. For example, it is well known from PET-scans that aggressive tumors with poor prognosis are almost always glucose/energy metabolism active tumors^[203,204]^. Some cancer cells strongly prefer to use glucose as the energy and carbon sources. These cancer cells are “addicted” to glucose and are more sensitive to glucose concentration changes than normal cells, dying significantly faster than normal cells under glucose deprivation^[99,166,167,205]^. In those cancer cases, using a glucose transport inhibitor or a glycolysis inhibitor in combination with another target drug may be particularly effective in inducing cancer cell death.

New cancer research indicates that TME plays very important roles in tumorigenesis and drug resistance. Any new therapies that expect to significantly improve the therapeutic outcomes must consider TME - tumor interactions. Intratumoral extracellular ATP has emerged as one of TME molecules that exert profound impacts on tumor cells regarding cell growth, survival, drug resistance and even metastasis^[161,179,181]^. Although different molecules can be used as the energy source for synthesizing ATP, the ultimate energy molecule used in all cells is ATP. Cancer cells appear to have to have higher ATP levels for survival and drug resistance. This difference between cancer and normal cells can be explored to combat cancer growth. If a method, such as tumor-selective targeting, can be developed and used to deliver ATP synthesis-inhibitor or extracellular ATP degrader resulting in reduction of ATP internalization inside tumors, tumors will be deficient in ATP, forcing them to stop growing or even undergo cell death. Extracellular ATP degradation or the inhibition of extracellular ATP internalization can be considered for combinational therapy with chemotherapeutics to enhance the anticancer efficacy of TKIs and chemo drugs. It is also conceivable that alteration of extracellular ATP levels may further enhance cancer immunotherapy, as immune cells in tumors are sensitive to ATP levels^[188-193]^.

Finally, the earlier the tumor is detected, the lower the heterogeneity of tumor cells would be, and the less drug resistance and the more successful the therapy should be. Early prevention and early detection should be considered with at least equal importance as cancer treatment at advanced stages.

## Declarations

### Data source and availability

Corresponding author may be contacted for any data inquiries.

### Copyright

© The Author(s) 2019.
